# Ectodysplasin A Is Increased in Non-Alcoholic Fatty Liver Disease, But Is Not Associated With Type 2 Diabetes

**DOI:** 10.3389/fendo.2021.642432

**Published:** 2021-03-04

**Authors:** Jacqueline Bayliss, Geraldine J. Ooi, William De Nardo, Yazmin Johari Halim Shah, Magdalene K. Montgomery, Catriona McLean, William Kemp, Stuart K. Roberts, Wendy A. Brown, Paul R. Burton, Matthew J. Watt

**Affiliations:** ^1^Department of Anatomy and Physiology, The University of Melbourne, Melbourne, VIC, Australia; ^2^Department of Surgery, Centre for Obesity Research and Education, Monash University, Melbourne, VIC, Australia; ^3^Department of Anatomical Pathology, Alfred Health, Melbourne, VIC, Australia; ^4^Department of Gastroenterology, The Alfred Hospital and Monash University, Melbourne, VIC, Australia

**Keywords:** ectodysplasin A, insulin resistance, hepatokine, type 2 diabetes (T2DM), non-alcoholic fatty liver disease

## Abstract

Ectodysplasin A (EDA) was recently identified as a liver-secreted protein that is increased in the liver and plasma of obese mice and causes skeletal muscle insulin resistance. We assessed if liver and plasma EDA is associated with worsening non-alcoholic fatty liver disease (NAFLD) in obese patients and evaluated plasma EDA as a biomarker for NAFLD. Using a cross-sectional study in a public hospital, patients with a body mass index >30 kg/m^2^ (n=152) underwent liver biopsy for histopathology assessment and fasting liver EDA mRNA. Fasting plasma EDA levels were also assessed. Non-alcoholic fatty liver (NAFL) was defined as >5% hepatic steatosis and nonalcoholic steatohepatitis (NASH) as NAFLD activity score ≥3. Patients were divided into three groups: No NAFLD (n=45); NAFL (n=65); and NASH (n=42). Liver *EDA* mRNA was increased in patients with NASH compared with No NAFLD (P=0.05), but not NAFL. Plasma EDA levels were increased in NAFL and NASH compared with No NAFLD (P=0.03). Plasma EDA was related to worsening steatosis (P=0.02) and fibrosis (P=0.04), but not inflammation or hepatocellular ballooning. ROC analysis indicates that plasma EDA is not a reliable biomarker for NAFL or NASH. Plasma EDA was not increased in patients with type 2 diabetes and did not correlate with insulin resistance. Together, we show that plasma EDA is increased in NAFL and NASH, is related to worsening steatosis and fibrosis but is not a reliable biomarker for NASH. Circulating EDA is not associated with insulin resistance in human obesity.

**Clinical Trial Registration:**

https://www.anzctr.org.au/Trial/Registration/TrialReview.aspx?ACTRN=12615000875505, identifier ACTRN12615000875505.

## Introduction

Non-alcoholic fatty liver disease (NAFLD) is now recognized as the most prevalent chronic liver disease worldwide (1). NAFLD is characterized by excessive hepatic triglyceride accumulation and is diagnosed as the presence of steatosis in >5% of hepatocytes, in the absence of significant alcohol consumption and other known causes of liver disease. Its more severe form, known as non-alcoholic steatohepatitis (NASH), is characterized by hepatic steatosis, inflammation and hepatocyte injury (*e.g.* ballooning), with or without hepatic fibrosis. NAFLD is present in ~25% of the general population (2) and incidence is increased strikingly in obesity, with overall NAFLD prevalence of ~75% and with 17% having NASH and/or steato-fibrosis (3). Patients with NAFLD also have a high prevalence of insulin resistance and several lines of evidence indicate a bidirectional relationship between NAFLD/NASH and type 2 diabetes (T2DM), perhaps owing to the shared mechanisms underpinning their pathology (4). Indeed, there is a high prevalence of NAFLD and NASH in patients with T2DM (~55%–80%) (5), while T2DM accelerates the progression of NAFLD and is an important clinical predictor of advanced hepatic fibrosis and mortality (6).

There is an incomplete understanding of the factors mediating the close relationship between NAFLD and diabetes, with the likelihood of multiple contributing factors. The secretion of liver-derived proteins, which are known as hepatokines, is altered in NAFLD (7) and a growing body of work shows that hepatokines signal *via* autocrine/paracrine and endocrine signaling to induce changes in lipid metabolism, peripheral insulin action, and glycemic control (8).

Ectodysplasin A (EDA) is a protein of the tumor necrosis factor family (9) that is encoded by the EDA gene. The *EDA* transcript is alternatively spliced producing different isoforms, with EDA-A1 and EDA-A2 being dominant and differing by only two amino acids. EDA is a type II membrane protein that can be cleaved by furin to produce a secreted form (10–12), which is subsequently recognized by the ectodysplasin A receptor (10, 13–15). EDA acts as a homotrimer and plays an important role in the development of ectodermal tissues such as skin (16, 17). EDA, along with c-Met, has also been shown to be involved in the differentiation of anatomical placodes, precursors of scales, feathers and hair follicles in vertebrates (18). Defects in this gene are a cause of X-linked hypohidrotic ectodermal dysplasia (19) and non-syndromic hypodontia (20).

Ectodysplasin A (EDA) was recently identified as a liver-secreted protein that appears to cause metabolic dysfunction in rodents (21). Gain- and loss- of-function studies showed that liver-derived EDA-A2 contributes to impaired skeletal muscle insulin sensitivity (21) and reduced liver triglyceride levels in obese, insulin resistant mice (22). Results from a small clinical study further reported increased liver EDA mRNA levels with increasing severity of steatosis and inflammation, and a negative correlation between liver EDA mRNA and whole-body insulin sensitivity (21). In addition, serum EDA-A2 levels were shown to be increased in overweight NAFLD patients compared with lean individuals, and showed positive associations between EDA-A2 and impaired glycemic control and inflammation (22). While these studies support a role for liver-derived EDA in the development of metabolic dysfunctions commonly associated with NAFLD, the clinical results are confounded by marked differences in adiposity between patient groups. Furthermore, there is an incomplete understanding of the relationship between EDA levels and the severity of liver disease in NAFLD, particularly in obesity, where the rate of any degree of NAFLD is markedly increased compared with lean individuals.

In light of these observations, the aims of the present study were to i) determine the role of EDA in progressive NAFLD as assessed by severity of steatosis, inflammation, ballooning, and fibrosis in NAFLD by liver histology, ii) assess the utility of EDA as a biomarker of NAFLD and, iii) determine the relationships between EDA, insulin resistance and other hepatic and systemic measures of lipid and glucose metabolism, and inflammation.

## Materials and Methods

### Ethics

Ethics was obtained from The Alfred Human Ethics Committee and the study was registered with the Australian Clinical Trials Register (ACTRN12615000875505). Patients were recruited from the following hospitals: The Alfred (195/15), The Avenue Hospital (190), and Cabrini Health (09-31-08-15) in Melbourne, Australia. All procedures were in accordance with the ethical standards of the Helsinki declaration.

### Patient Recruitment

Patients were selected and recruited from individuals undergoing laparoscopic adjustable gastric band, sleeve gastrectomy or gastric bypass surgery. Inclusion criteria were as follows: 1) > 18 years of age, 2) BMI ≥ 30 kg/m^2^ and 3) an elevated alanine aminotransferase (ALT), aspartate aminotransferase (AST) or gamma-glutamyl transferase (GGT) value that was 0.5 times higher than the upper normal limit. Patients were excluded from the study if they showed evidence of having another liver disease including viral hepatitis or history of excess alcohol use, defined by the American Association for the Study of Liver Disease (23).

### Clinical and Biochemical Data

On the day of the operation patients underwent a full physical examination and medical history, including a full list of current medication. Fasting (8–12 h) blood samples were collected before the induction of anesthesia. Blood was collected into tubes containing K_2_EDTA or SST™ II advance tubes, centrifuged for 10 min at 1792 x *g* and plasma was collected and stored at -80°C for subsequent analysis.

### Liver Biopsy, Tissue Collection, and Histopathology

A liver wedge biopsy, at least 1 cm in depth, was collected from patients. The sample was divided into two pieces where half was placed into 10% formalin and stained with hematoxylin and eosin and Masson’s trichrome for subsequent histological assessment. The other half was snap frozen and stored at -80°C for later analysis.

Biopsies were evaluated in a blinded fashion by a pathologist. This was based on the presence or absence of the following three components: i) steatosis (5%–33% of parenchyma for grade 1, >33% to 66% for grade 2, and >66% for grade 3); ii) lobular inflammation (<2 foci per ×200 field for grade 1, 2–4 foci for grade 2, and >4 foci for grade 3); and iii) hepatocellular ballooning where few or many ballooning cells are present per high-power field for grade 1 or 2, respectively (24). NASH was determined as the joint presence of steatosis, ballooning, and lobular inflammation (NAFLD activity score ≥3) as defined by the Clinical Practice Guidelines of European Association for the Study of the Liver (EASL), the European Association for the Study of Diabetes (EASD) and European Association for the Study of Obesity (EASO) (25). Liver fibrosis was graded according to the Kleiner classification (26).

### Analysis of Plasma EDA

Human plasma samples were analyzed by colorimetric assay for ectodysplasin A using the Human Ectodysplasin A (EDA) ELISA Kit (My BioSource, San Diego, CA, USA). The inter-assay coefficient of variation was 8.46.

### Analysis of Liver EDA mRNA

RNA from the liver biopsies was extracted using TRI-Reagent (Sigma-Aldrich, Castle Hill, NSW, Australia) following the manufacturers protocol. Following extraction, the RNA was incubated with a DNA removal kit (Invitrogen, Carlsbad, California, United States), following the manufacturer’s instructions. After RNA quantification, synthesis of complementary DNA was performed using an iSCRIPT kit (Bio-Rad, Hercules, California, United States). Real Time PCR was performed using SYBR Green Master Mix (Qiagen, Hilden, Germany), respective primers and read using a CFX384 Touch Real-Time PCR Detection System (Biorad, Hercules, California, United States). Primers used: EDA Forward: GGACGGCACCTACTTCATCTA and Reverse: GCGGTATAGCAAGTGTTGTAGTT. The housekeeping gene used to normalize values was hypoxanthine–guanine phosphoribosyltransferase (*HPRT*), primer sequences; Forward: ATAAGCCAGACTTTGTTGG and Reverse: ATAGGACTCCAGATGTTTCC.

### Statistical Analysis

A two-sided p value of 0.05 was considered statistically significant. A Shapiro-Wilk test was used to assess normality of distribution. Continuous parametric variables were represented as mean ± standard deviation (SD) and compared using student t-test or one-way ANOVA. Median ± 95% confidence interval were used to described non-parametric variables. Comparative analysis was performed using Mann-Whitney U test or Kruskal-Wallis test. Binary data was reported as whole numbers and percentage and compared using Chi-square test. Univariate binary and linear logistic regressions determined the relationship between each variable and the cohorts, and with plasma EDA respectively. Statistically significant variables from the univariate analysis were further evaluated using multivariate regression with stepwise backward (Wald). Omnibus tests of model coefficients were used to determine overall model fit and statistical significance. Nagelkerke *R^2^* method was used to determine how much variation can be explained by the model. The receiver operating characteristic (ROC) curves were performed to determine the threshold of plasma EDA that distinguish NASH and NAFLD from no NAFLD. The area under the curve (AUC) represents the overall discriminatory ability of the ROC curve. AUCs were classified according to Hosmer et al, where AUC more than 0.9 was considered outstanding, between 0.8 and 0.9 excellent, between 0.7 and 0.8 acceptable, and less than 0.7 was poor discrimination (27). Statistical analysis was performed with SPSS version 26 (SPSS Inc, Chicago, IL, USA) and GraphPad Prism version 8.3.0 (GraphPad Software, San Diego, California USA).

## Results

### Patient Characteristics

The patient’s clinical characteristics are shown in **Table 1** and liver pathology in **Table 2**. Samples were collected and analyzed from 152 patients with a strong selection bias for female patients (75%). The average age of the patient cohort was 45 ± 3 years and the body mass index (BMI) averaged 47.8 ± 3.3 kg/m^2^. Liver histology identified 45 patients (29.6%) who exhibited no adverse pathology, which are from this point forward referred to as ‘No NAFLD’. The other patients are classified as NAFL (65 patients, 42.8%) or NASH (42 patients, 27.6%) based on a widely used pathology score (26). Consistent with the histopathology assessment, there was a significant increase in circulating levels of the liver enzymes AST and ALT in NASH compared with No NAFLD and NAFL (P<0.001). No differences between groups were observed for age, BMI and blood lipids with the exceptions of plasma triglyceride, which was increased in NAFL and NASH compared with No NAFLD (P=0.002), and plasma HDL, which was decreased in NASH compared with No NALFD (P=0.004). Patients with NAFL and NASH were insulin resistant compared with No NAFLD, as indicated by an increased HOMA-IR (P=0.005) (**Table 1**), while type 2 diabetes prevalence was not different between groups (P=0.124).

**Table 1:** Clinical and biochemical characteristics of subjects.

	No NAFLD (n=45)	NAFL (n=65)	NASH (n=42)	*p-value*
Males (n, %)	8 (17.8%)	14 (21.5%)	16 (38.1%)	0.064^*^
BMI (kg/m^2^)	41.9 (8.9)	42.9 (8.8)	44.6 (11.5)	0.127^^^
Age (years)	46.0 (27.5)	44.5 (20.3)	47.0 (17.0)	0.719^^^
Patients with T2DM (n, %)	6 (13.3%)	15 (23.1%)	13 (31.7%)	0.124^*^
**AST (IU/L)**	**23.0 (10.5)**	**25.5 (10.0)**	**33.0 (25.0)^b,c^**	**<0.001^^^**
**ALT (IU/L)**	**26.0 (18.5)**	**30.5 (15.3)**	**46.0 (26.0)^b,c^**	**<0.001^^^**
**GGT (IU/L)**	**22.0 (16.0)**	**34.5 (24.5)^a^**	**35.0 (18.0)^b^**	**0.012^^^**
ALP (IU/L)	70.1 ± 19.1	74.6 ± 21.1	69.5 ± 19.1	0.985^#^
**Triglyceride (mmol/L)**	**1.1 (0.6)**	**1.3 (0.7)^a^**	**1.4 (0.8)^b^**	**0.002^^^**
Total cholesterol (mmol/L)	3.9 (1.1)	3.9 (1.5)	4.1 (1.4)	0.406**^^^**
**HDL (mmol/L)**	**1.0 (0.3)**	**0.9 (0.3)**	**0.9 (0.3)^b^**	**0.004^^^**
LDL (mmol/L)	2.3 ± 0.8	2.3 ± 0.9	2.5 ± 0.6	0.361^#^
Fasting blood glucose (mmol/L)	5.1 (1.5)	5.2 (1.1)	5.7 (1.9)	0.086**^^^**
Hba1c (%)	5.6 (0.5)	5.7 (1.2)	5.9 (2.4)	0.075**^^^**
Insulin (mU/L)	5.1 (5.8)	6.8 (8.3)	8.1 (10.7)	0.067**^^^**
**C-peptide (pmol/L)**	**600 (388)**	**797 (570)**	**928 (504)^b^**	**0.014^^^**
**HOMA-IR**	**0.59 (0.69)**	**1.05 (1.35)^a^**	**1.10 (1.38)^b^**	**0.005^^^**
Urea (mmol/L)	4.3 (1.8)	4.4 (2.2)	4.6 (2.0)	0.986**^^^**
Creatine (µmol/L)	63.0 (11.0)	64.0 (13.3)	70.0 (19.5)	0.306**^^^**
eGFR (ml/min/1.73 m²)	90.0 (0)	90.0 (2.25)	90.0 (0)	0.438**^^^**
Albumin (g/L)	35.0 (6.0)	36.0 (5.3)	36.0 (4.0)	0.349**^^^**
Bilirubin (mmol/L)	8.0 (6.0)	8.0 (5.5)	8.0 (7.0)	0.743**^^^**
White Cell count (x10^9^)	6.7 (2.8)	7.3 (3.7)	7.9 (3.0)	0.269**^^^**
Platelets (x10^9^)	230 ± 58	253 ± 55	238 ± 62	0.290^#^

*Chi-square test, ^Kruskal-Wallis Test with pairwise comparisons (Bonferroni correction), ^#^One-way ANOVA, ^a^p<0.05 no NAFLD vs. NAFL, ^b^p<0.05 no NAFLD vs. NASH, ^c^p<0.05 NAFL vs. NASH. Numbers in bold are significant. Data are shown as mean ± SD for continuous variables and % for categorial variables. NAFLD, Non-alcoholic fatty liver disease; NASH, Non-alcoholic steatohepatitis; BMI, Body Mass Index; AST, Aspartate aminotransferase; ALT, Alanine aminotransferase; GGT, Gamma glutamyl transferase; HDL, High Density Lipoprotein; LDL, Low Density Lipoprotein; ALP, Alkaline phosphatase; Hba1c, Hemoglobin A1c; HOMA-IR, Homeostatic Model Assessment of Insulin Resistance; and eGFR, Estimated Glomerular Filtration Rate.

**Table 2:** Liver pathology in human subjects.

	No NAFLD (n=45)	NAFL (n=65)	NASH (n=42)
**Steatosis Grade % (n, %)**			
0 – <5	45 (100%)	0 (0%)	0 (0%)
1 – 5–33	0 (0%)	40 (61.5%)	6 (14.3%)
2 – 34–66	0 (0%)	24 (37%)	25 (59.5%)
3 – > 66	0 (0%)	1 (1.5%)	11 (26.2%)
**Lobular inflammation (n, %)**			
0 – none	43 (95.6)	47 (72.3%)	0 (0%)
1 – <2	2 (4%)	16 (24.6%)	39 (92.9%)
2 – 2–4	0 (0%)	2 (3.1%)	3 (7.1%)
3 – >4	0 (0%)	0 (0%)	0 (0%)
**Ballooning (n, %)**			
0 – none	45 (100%)	56 (86.2%)	0 (0%)
1 – few	0 (0%)	6 (9.2%)	33 (78.6%)
2 – many	0 (0%)	3 (4.6%)	9 (21.4%)
**Fibrosis (n, %)**			
0 – none	45 (100%)	57 (87.7%)	14 (33.3%)
1 – Perisinusoidal or periportal	0 (0%)	5 (7.7%)	26 (61.9%)
2 – Perisinusoidal and portal/periportal fibrosis	0 (0%)	1 (1.5%)	2 (4.8%)
3 – Bridging	0 (0%)	1 (1.5%)	0 (0%)
4 – Cirrhosis	0 (0%)	1 (1.5%)	0 (0%)

### Relationship of Liver EDA mRNA With NAFLD

**Figure 1:**
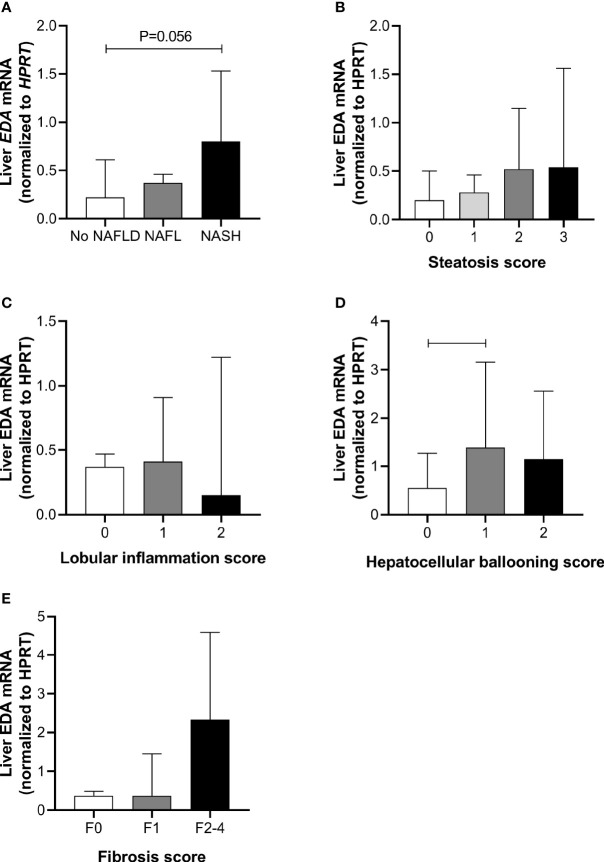
Liver EDA mRNA and NAFLD progression. Hepatic *EDA* expression by **(A)** patient group, **(B)** steatosis grade, **(C)** lobular inflammation score, **(D)** hepatocellular ballooning score and **(E)** fibrosis score. Shown are median and 95% confidence interval. Data were analyzed by Kruskal-Wallis test and Dunn’s multiple comparisons test. Adjoining lines indicate P<0.05.

Liver *EDA* mRNA was not increased in NAFL compared with No NAFLD, but appeared increased in NASH compared with No NAFLD (P=0.054) (**Figure 1A**). There was no difference in liver *EDA* mRNA between No NAFLD and NAFLD (P=0.12). Liver *EDA* mRNA tended to increase with worsening grades of steatosis (P=0.06 by ANOVA) (**Figure 1B**) but were not impacted by lobular inflammation (P=0.32) (**Figure 1C**). Liver *EDA* mRNA was higher in patients with hepatocellular ballooning score > 0, although this was statistically significant when comparing scores of 0 *vs.*1, but not 0 *vs.* 2 (**Figure 1D**). Liver *EDA* mRNA was not different (P=0.83) when patients were stratified for no fibrosis (F0), mild fibrosis (F1) or moderate to advanced fibrosis (F0 *vs.* F2–4) (**Figure 1E**).

### Relationship of Plasma EDA With Progressive NAFLD

**Figure 2:**
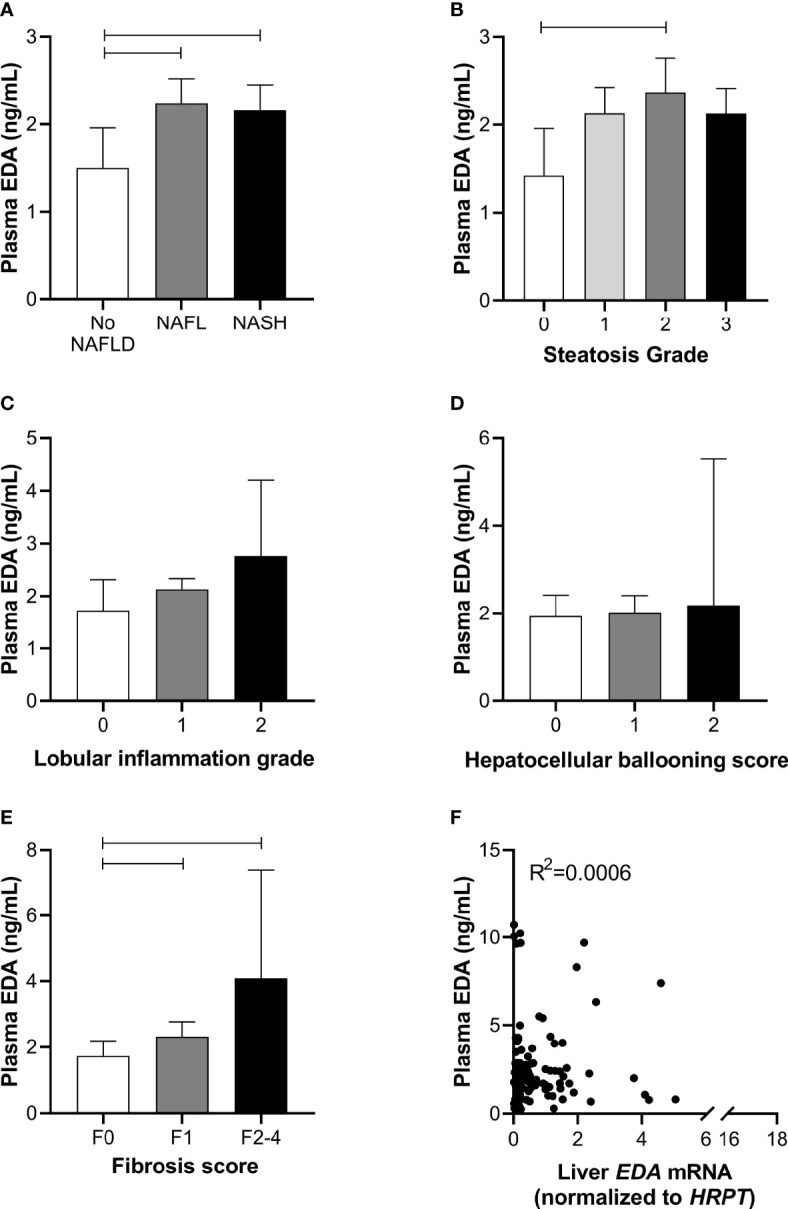
Plasma EDA and NAFLD progression. Plasma EDA levels by **(A)** patient group, **(B)** steatosis grade, **(C)** lobular inflammation score, **(D)** hepatocellular ballooning score and **(E)** fibrosis score. Shown are median and 95% confidence interval. Data were analyzed by Kruskal-Wallis test and Dunn’s multiple comparisons test. Adjoining lines indicate P<0.05. **(F)** Relationship between liver *EDA* mRNA and plasma EDA.

To further assess the role of EDA in relation to progressive NAFLD in obesity, we analyzed plasma EDA levels by ELISA. Plasma EDA concentrations averaged 2.47 ± 0.17 ng/ml in the entire patient cohort and were increased by 55% in NAFL and 52% in NASH when compared with No NAFLD (P = 0.03) (**Figure 2A**). Plasma EDA was increased with steatosis grade above 0 (P=0.04 by ANOVA), reaching significance when comparing grade 0 *vs.* grade 2 (**Figure 2B**). Plasma EDA was not related to the degree of inflammation (P=0.40) or hepatocellular ballooning (P=0.61), but was increased with the severity of fibrosis (P=0.007) (**Figure 2C–E**). EDA was recently identified as a hepatokine (21). Accordingly, we assessed the relationship between liver *EDA* mRNA and plasma EDA and report no significant correlation between liver *EDA* mRNA and plasma EDA (R^2 =^ 0.0006, P=0.79) (**Figure 2F**).

**Figure 3:**
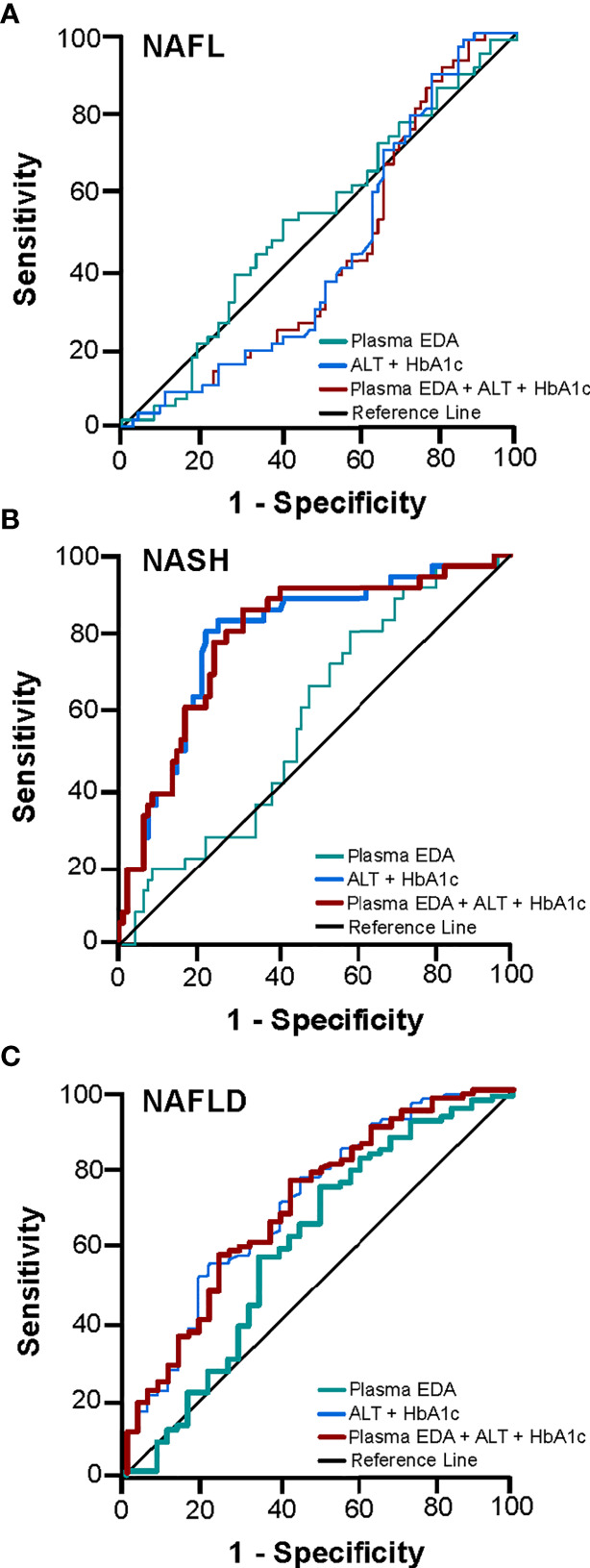
Receiver operating characteristic (ROC) curve for the prediction of NAFL and NASH. The diagnostic accuracy of clinical markers with and without the addition of EDA was calculated for **(A)** NAFL, **(B)** NASH, and **(C)** NAFLD. Thicker lines represent statistically significant models with P<0.05.

We next explored the potential utility of plasma EDA as a biomarker of NAFLD. ROC curves were developed to predict the presence of NAFL or NASH. Area under ROC was 0.611 for NAFL (95% CI 0.497–0.725, P=0.054; **Figure 3A**) and 0.569 for NASH (95% CI 0.470–0.667, P=0.203; **Figure 3B**). When comparing No NAFLD and NAFLD (e.g. NAFL and NASH combined), the AUC was 0.623 (95% CI 0.514–0.731, P=0.021; **Figure 3C**). Plasma EDA of above or equal to 1.423 ng/ml predicts NAFLD with the sensitivity of 74.6% and specificity of 50%.

The diagnostic accuracy of clinical markers with and without the addition of EDA was calculated. Multivariable analysis identified HbA1c and ALT as statistically significant variables for the presence of NASH and NAFLD. A combination of HbA1c and ALT produced an AUROC for NASH of 0.794 (95% CI 0.706–0.882, P<0.001) and for NAFLD of 0.711 (95% CI 0.611–0.810, P<0.001) (**Figures 3B, C**). Combination of these clinical markers with plasma EDA produced an AUROC for NASH of 0.793 (95% CI 0.705–0.881, P<0.001) and for NAFLD 0.706 (95% CI 0.607–0.805, P<0.001), which was not a significant improvement compared to routine variables alone (**Figures 3B, C**). Combinations of established scoring systems (NAFLD liver fat score and hepatic steatosis index) and plasma EDA with or without the addition of HbA1c and ALT did not improve the accuracy of these scoring system to diagnose NAFLD or NASH above HbA1c and ALT alone (data not shown). Further, plasma EDA, clinical markers, established scoring systems, and combinations of these variables failed to produce statistically significant ROC to diagnose NAFL (**Figure 3A**).

### Relationship of Plasma EDA With Insulin Resistance and Type 2 Diabetes

**Figure 4:**
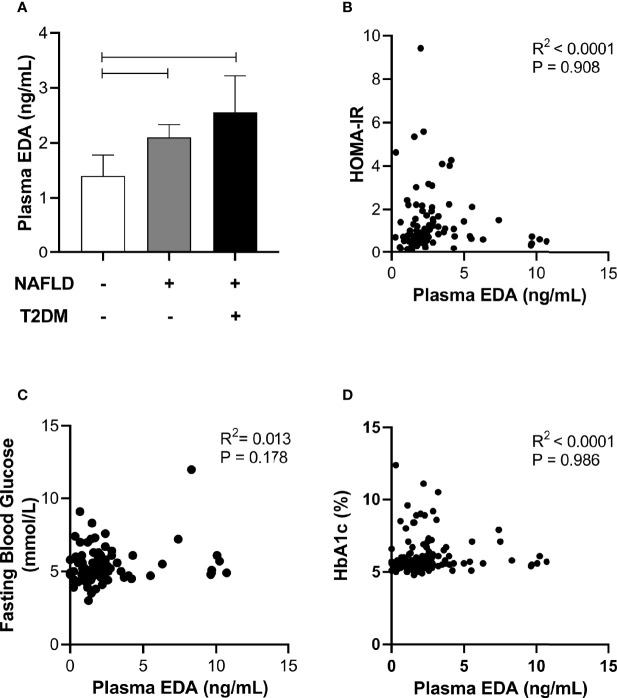
Relationship between plasma EDA, type 2 diabetes and insulin resistance. **(A)** Plasma EDA in patients without NAFLD or type 2 diabetes, with NAFLD and without type 2 diabetes and with both NAFLD and type 2 diabetes. Data were analyzed by Kruskal-Wallis test and Dunn’s multiple comparisons test. Adjoining lines indicate P<0.05. **(B–D)** Relationship between plasma EDA and **(B)** HOMA-IR (insulin resistance), **(C)** fasting blood glucose, and **(D)** HbA1c.

Circulating EDA is proposed to contribute to insulin resistance in skeletal muscle through endocrine regulation (21). Accordingly, we assessed plasma EDA levels in patients without and with type 2 diabetes that were matched for NAFLD. Plasma EDA was increased in patients with NAFLD compared with No NAFLD but was not significantly different between NAFLD patients with and without type 2 diabetes (**Figure 4A**). We also assessed the relationship between plasma EDA and measures of insulin sensitivity and glycemic control in the entire patient cohort. Plasma EDA was not significantly correlated with whole body insulin resistance determined using the HOMA-IR (R²<0.0001, P=0.908), fasting plasma glucose (R²=0.013, P=0.178), or HbA1c (R^2^<0.0001, P=0.986) (**Figures 4B–D**). These data indicate that plasma EDA is not associated with insulin sensitivity or type 2 diabetes in obese individuals. Moreover, univariate binary regression analysis revealed no significant associations of plasma EDA with any clinical measure (**Table 3**).

**Table 3:** Association of plasma EDA with clinical measures.

	Univariate regression			
	R^2^	Beta	95% CI of Beta	p-value
Males	<0.0001	0.011	-0.810–0.832	0.979
BMI	0.105	0.028	-0.016–0.072	0.207
T2DM	0.006	0.388	-0.459–1.235	0.366
Age	0.013	0.020	-0.009–0.048	0.179
AST	0.017	0.010	-0.002–0.022	0.117
ALT	0.011	0.006	-0.003–0.015	0.211
GGT	<0.0001	-0.001	-0.011–0.009	0.849
ALP	0.013	0.012	-0.005–0.028	0.181
Triglyceride	0.013	0.335	-0.0148–0.818	0.172
Total cholesterol	0.001	0.084	-0.292–0.460	0.661
HDL	0.003	-0.472	-1.865–0.921	0.504
LDL	0.001	0.073	-0.365– -.512	0.742
Fasting blood glucose	0.013	0.118	-0.054–0.290	0.178
HbA1c	<0.0001	-0.002	-0.276–0.271	0.986
C-peptide	0.020	0.001	0.000–0.001	0.101
HOMA-IR	<0.0001	0.016	-0.259–0.292	0.908
Insulin	0.001	0.004	-0.015–0.023	0.669
Urea	0.026	0.190	-0.003–0.383	0.054
Creatinine	0.007	0.008	-0.008–0.025	0.313
eGFR	0.023	-0.036	-0.075–0.003	0.069
Albumin	0.001	0.019	-0.069–0.107	0.666
Bilirubin	0.001	-0.012	-0.076–0.053	0.724
White cell count	0.007	-0.073	-0.214–0.069	0.313
Platelet	0.004	-0.002	-0.009–0.004	0.436

Multivariate analysis not performed because there were no statistically significant factors identified in the univariate analysis.

## Discussion

The liver is an active endocrine organ and is susceptible to substantial metabolic and inflammatory stress in obesity, factors which have been shown to mediate changes in liver-secreted proteins (*i.e.* hepatokines) (7, 8). Accordingly, hepatokines have been identified for their potential use as biomarkers across the spectrum of NAFLD (28). This study focused on understanding the links between the hepatokine ectodysplasin A, NAFLD and NAFLD co-morbidities in obese individuals.

At present, the only reliable method of identifying and staging patients with NAFLD is liver biopsy; however, this procedure is invasive, is associated with sampling variability and limited representation of the whole liver and is difficult to repeat to monitor progression of liver damage. With the increasing global incidence of obesity and associated NAFLD (3) and the lack of diagnostic precision with alternative non-invasive assessments for NAFLD in obese patients (*e.g.* plasma biomarkers and elastography techniques) (29), there is an urgent need for non-invasive alternatives to liver biopsy to identify those patients who require intervention and to monitor therapeutic responses in these patients. The identification of accurate biomarkers has, to some degree, been hampered by the lack of clarity in understanding the mechanisms mediating NAFLD development and progression (30).

We report increased levels of plasma EDA in obese individuals with NAFL compared with obese individuals with no adverse liver pathology, but no further increase in NASH subjects. Consistent with this observation, plasma EDA was increased with steatosis grade but not with grade of lobular inflammation or hepatocellular ballooning and was not related to other measures of liver damage including AST and GGT. Extending on these findings, we used receiver operating characteristic regression to calculate the area under the ROC curve for plasma EDA alone and in combination with other diagnostic tests to investigate the diagnostic accuracy of EDA for the prediction of NAFLD. Plasma EDA demonstrated limited clinical utility alone and did not improve the diagnostic accuracy of clinical routine markers (HbA1c and ALT) and established scoring systems (NFS and hepatic steatosis index) for NAFLD or NASH. Collectively, our analysis in a large cohort of obese patients with well-defined liver histopathology supports the notion that EDA is increased in NAFLD but is an unreliable biomarker for NAFLD or discriminator of NAFL and NASH. It is noted that a very low number of subjects had significant fibrosis (F3–4) and therefore the role of EDA in patients with advanced form for NAFLD cannot be excluded. From a broader perspective, the use of biomarkers is problematic when attempting to discriminate between patients with NAFL, NASH and fibrosis because this is a dynamic disease in which the worsening and partial amelioration of both conditions is reported to occur (31, 32).

A previous study in a cohort of lean individuals reported a strong association between NAFLD and circulating EDA-A2, with the authors concluding that EDA-A2 is a useful biomarker for NAFLD (22). There were significant differences between studies. The previous study by Yang et al. (22) determined NAFLD using ultrasonography and graded NAFLD according to the Chinese Standard (33), whereas in the present study NAFLD was determined using liver biopsy and histological assessment according to the guidelines of the Clinical Practice Guidelines of European Association for the Study of the Liver (25). The second major difference was the patient cohort, with Yang et al. (22) examining a lean cohort compared with our investigation of EDA in morbidly obese patients undergoing bariatric surgery. This raises the possibility that plasma EDA is a useful biomarker in lean patients, but not in obese patients where NAFLD incidence is significantly higher. The third major difference is that Yang et al. (22) assessed plasma EDA-A2 whereas we assessed total plasma EDA. The *EDA* transcript is alternatively spliced producing nine different isoforms, with EDA-A1 and EDA-A2 being dominant and differing by only two amino acids (34). We were unable to confirm the specificity of the EDA-A2 ELISA used in the previous study (22) and given the extremely high homology between EDA-A1 and EDA-A2, elected to measure total EDA. To the best of our knowledge, the ratio between EDA-A1 and -A2 in human plasma is unknown and this should be the focus of future investigations.

**Figure 5:**
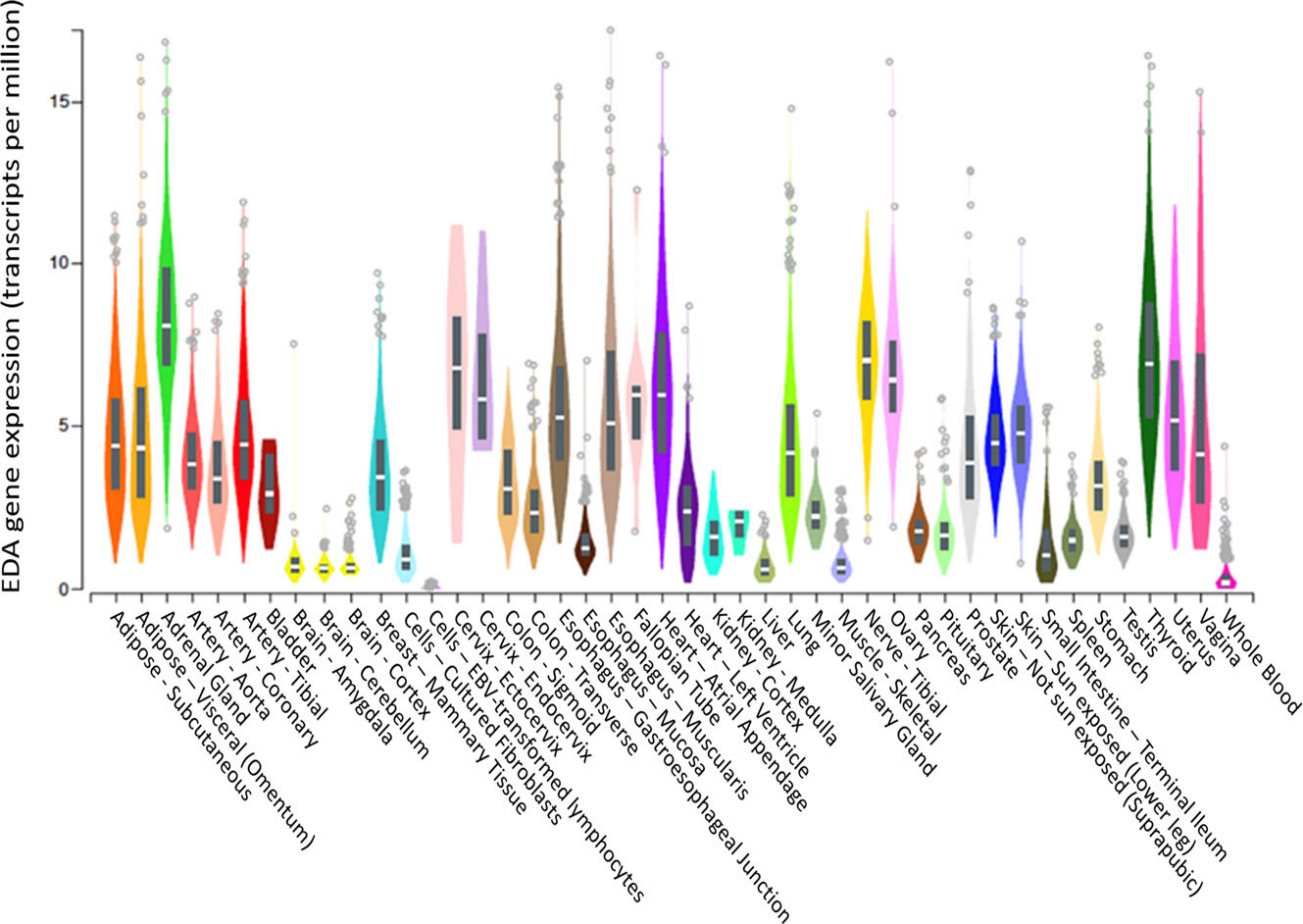
*EDA* gene expression in various tissues. *EDA* tissue gene expression (shown as TPM—transcripts per million) adapted from GTEx (based on ENSG00000158813), showing that the liver is the tissue with the 6^th^ highest *EDA* expression compared to 54 tissues examined. Box plots are shown as median and 25^th^/75^th^ percentile. Data Source: GTEx Analysis Release V8 (dbGaP Accession phs000424.v8.p2).

Although EDA was previously and convincingly identified as a hepatokine in mice (21), we did not observe a significant correlation between liver *EDA* mRNA expression and plasma EDA levels, suggesting that the liver might not be a major contributor to circulating EDA levels in humans. In this respect, Awazawa and colleagues (21) reported expression of *Eda* mRNA in murine white and brown adipose tissue, skeletal muscle, heart, brain and liver of mice, with the highest expression in brown fat and the lowest expression in the liver. These observations are corroborated by the Genotype-Tissue Expression (GTEx) project showing low *EDA* mRNA expression in liver and high expression in visceral adipose tissue and pancreas (**Figure 5**). Hence, it is probable that tissues other than the liver are the major contributors to circulating EDA levels in humans, with white adipose tissue a logical candidate. Notably, there were no correlations between BMI (a marker of total adiposity) and plasma EDA in our study. It is possible that other factors contribute to the mismatch between liver *EDA* mRNA and circulating EDA. For instance, EDA requires cleavage by the endopeptidase furin to facilitate protein secretion (11) and differential furin expression might contribute to changes in liver EDA secretion in progressive NAFLD.

NAFLD is closely linked to several co-morbidities including insulin resistance and type 2 diabetes. Liver *EDA* mRNA was shown to correlate with systemic insulin resistance in patients and loss and gain of function studies in mice ascribed a role for liver-derived EDA in the development of skeletal muscle insulin resistance (21, 22). Our data do not support these findings, as neither liver nor plasma EDA were significantly associated with markers of insulin resistance (*i.e.* HOMA-IR, fasting glucose and fasting insulin) or type 2 diabetes. Our study has several limitations in relation to these previous findings; the ELISA used in this study detects EDA-A1 and EDA-A2 isoforms which does not allow for assessment of EDA-A2 as a mediator of insulin resistance. Furthermore, we do not have measures of skeletal muscle insulin resistance in our patient cohort, which would require assessment with euglycemic-hyperinsulinemic clamps.

In conclusion, we show in a large obese patient cohort with liver biopsy-defined NAFLD stage and extensive blood profiling that liver *EDA* expression and plasma EDA concentration are increased in NAFL and NASH, but that circulating EDA is not a reliable biomarker for NAFLD. Circulating EDA did not correlate with clinical measures of liver damage, fat mass, blood lipids, insulin resistance or type 2 diabetes. Finally, we find no correlation between liver and plasma EDA levels, suggesting that the liver is unlikely to be a major contributor to circulating EDA in humans.

## Data Availability Statement

The raw data supporting the conclusions of this article will be made available by the authors, without undue reservation.

## Ethics Statement

The studies involving human participants were reviewed and approved by The Alfred (195/15), Avenue (190) and Cabrini (09-31-08-15) Human Research Ethics Committees. This study was registered with the Australian Clinical Trials Register (ACTRN12615000875505). The patients/participants provided their written informed consent to participate in this study.

## Author Contributions

GO, WK, SR, WB, PB, and MW contributed to study design. GO, WB, and PB collected patient tissues. CM performed histopathology. JB analyzed data. WN and YS performed statistical analyses. JB, MM, and MW drafted the manuscript. All authors contributed to the article and approved the submitted version.

## Funding

These studies were supported by the National Health and Medical Research Council of Australia (NHMRC, APP1162511). MM is supported by Research Fellowships from the NHMRC (APP1143224). WN is supported by a Melbourne Research Scholarship (University of Melbourne).

## Conflict of Interest

MW has received consultancy fees from Gilead Science, Inc. WB has received grants from Johnson and Johnson, Medtronic, GORE, Applied Medical, and Novo Nordisk, and personal fees from GORE, Novo Nordisk, and Merck Sharpe and Dohme for lectures and advisory boards. All were outside the submitted work.

The remaining authors declare that the research was conducted in the absence of any commercial or financial relationships that could be construed as a potential conflict of interest.

## References

[1] Younossi ZM Stepanova M Afendy M Fang Y Younossi Y Mir H. Changes in the prevalence of the most common causes of chronic liver diseases in the United States from 1988 to 2008. Clin Gastroenterol Hepatol Off Clin Pract J Am Gastroenterol Assoc (2011) 9(6):524–30.e1; quiz e60. 10.1016/j.cgh.2011.03.020 21440669

[2] Younossi ZM Koenig AB Abdelatif D Fazel Y Henry L Wymer M. Global epidemiology of nonalcoholic fatty liver disease-Meta-analytic assessment of prevalence, incidence, and outcomes. Hepatol (Baltimore Md) (2016) 64(1):73–84. 10.1002/hep.28431 26707365

[3] Ooi GJ Burton PR Bayliss J Raajendiran A Earnest A Laurie C. Effect of Body Mass Index, Metabolic Health and Adipose Tissue Inflammation on the Severity of Non-alcoholic Fatty Liver Disease in Bariatric Surgical Patients: a Prospective Study. Obes Surg (2019) 29(1):99–108. 10.1007/s11695-018-3479-2 30229460

[4] Ryysy L Hakkinen AM Goto T Vehkavaara S Westerbacka J Halavaara J. Hepatic fat content and insulin action on free fatty acids and glucose metabolism rather than insulin absorption are associated with insulin requirements during insulin therapy in type 2 diabetic patients. Diabetes (2000) 49(5):749–58. 10.2337/diabetes.49.5.749 10905483

[5] Younossi ZM Golabi P de Avila L Paik JM Srishord M Fukui N. The global epidemiology of NAFLD and NASH in patients with type 2 diabetes: A systematic review and meta-analysis. J Hepatol (2019) 71(4):793–801. 10.1016/j.jhep.2019.06.021 31279902

[6] Stepanova M Rafiq N Makhlouf H Agrawal R Kaur I Younoszai Z. Predictors of all-cause mortality and liver-related mortality in patients with non-alcoholic fatty liver disease (NAFLD). Digest Dis Sci (2013) 58(10):3017–23. 10.1007/s10620-013-2743-5 23775317

[7] Meex RC Hoy AJ Morris A Brown RD Lo JC Burke M. Fetuin B Is a Secreted Hepatocyte Factor Linking Steatosis to Impaired Glucose Metabolism. Cell Metab (2015) 22(6):1078–89. 10.1016/j.cmet.2015.09.023 26603189

[8] Watt MJ Miotto PM De Nardo W Montgomery MK. The Liver as an Endocrine Organ-Linking NAFLD and Insulin Resistance. Endocr Rev (2019) 40(5):1367–93. 10.1210/er.2019-00034 31098621

[9] Mikkola ML Pispa J Pekkanen M Paulin L Nieminen P Kere J. Ectodysplasin, a protein required for epithelial morphogenesis, is a novel TNF homologue and promotes cell-matrix adhesion. Mech Dev (1999) 88(2):133–46. 10.1016/S0925-4773(99)00180-X 10534613

[10] Elomaa O Pulkkinen K Hannelius U Mikkola M Saarialho-Kere U Kere J. Ectodysplasin is released by proteolytic shedding and binds to the EDAR protein. Hum Mol Genet (2001) 10(9):953–62. 10.1093/hmg/10.9.953 11309369

[11] Schneider P Street SL Gaide O Hertig S Tardivel A Tschopp J. Mutations leading to X-linked hypohidrotic ectodermal dysplasia affect three major functional domains in the tumor necrosis factor family member ectodysplasin-A. J Biol Chem (2001) 276(22):18819–27. 10.1074/jbc.M101280200 11279189

[12] Chen Y Molloy SS Thomas L Gambee J Bächinger HP Ferguson B. Mutations within a furin consensus sequence block proteolytic release of ectodysplasin-A and cause X-linked hypohidrotic ectodermal dysplasia. Proc Natl Acad Sci USA (2001) 98(13):7218–23. 10.1073/pnas.131076098 PMC34649 11416205

[13] Kowalczyk-Quintas C Schneider P Ectodysplasin A. (EDA) - EDA receptor signalling and its pharmacological modulation. Cytokine Growth factor Rev (2014) 25(2):195–203. 10.1016/j.cytogfr.2014.01.004 24508088

[14] Li M Bai YT Han K Li XD Meng J. Knockdown of ectodysplasin-A receptor-associated adaptor protein exerts a tumor-suppressive effect in tongue squamous cell carcinoma cells. Exp Ther Med (2020) 19(5):3337–47. 10.3892/etm.2020.8578 PMC7132229 32266031

[15] Schuepbach-Mallepell S Kowalczyk-Quintas C Dick A Eslami M Vigolo M Headon DJ. Methods for the Administration of EDAR Pathway Modulators in Mice. Methods Mol Biol (Clifton NJ) (2021) 2248:167–83. 10.1007/978-1-0716-1130-2_12 33185875

[16] Kere J Srivastava AK Montonen O Zonana J Thomas N Ferguson B. X-linked anhidrotic (hypohidrotic) ectodermal dysplasia is caused by mutation in a novel transmembrane protein. Nat Genet (1996) 13(4):409–16. 10.1038/ng0895-409 8696334

[17] Wang X Zhang Z Yuan S Ren J Qu H Zhang G. A novel EDA1 missense mutation in X-linked hypohidrotic ectodermal dysplasia. Medicine (2020) 99(11):e19244. 10.1097/MD.0000000000019244 32176048 PMC7220389

[18] Barrow-McGee R Kishi N Joffre C Ménard L Hervieu A Bakhouche BA. Beta 1-integrin-c-Met cooperation reveals an inside-in survival signalling on autophagy-related endomembranes. Nat Commun (2016) 7:11942. 10.1038/ncomms12392 27336951 PMC4931016

[19] Wohlfart S Meiller R Hammersen J Park J Menzel-Severing J Melichar VO. Natural history of X-linked hypohidrotic ectodermal dysplasia: a 5-year follow-up study. Orphanet J rare Dis (2020) 15(1):7. 10.1186/s13023-019-1288-x 31924237 PMC6954509

[20] Al-Ani AH Antoun JS Thomson WM Topless R Merriman TR Farella M. Common variants of EDA are associated with non-syndromic hypodontia. Orthodontics craniofacial Res (2021) 24(1):155–63. 10.1111/ocr.12419 32772440

[21] Awazawa M Gabel P Tsaousidou E Nolte H Kruger M Schmitz J. A microRNA screen reveals that elevated hepatic ectodysplasin A expression contributes to obesity-induced insulin resistance in skeletal muscle. Nat Med (2017) 23(12):1466–73. 10.1038/nm.4420 29106399

[22] Yang J Zhou W Zhu J Wu Y Xu L Wang Y. Circulating ectodysplasin A is a potential biomarker for nonalcoholic fatty liver disease. Clinica chimica acta; Int J Clin Chem (2019) 499:134–41. 10.1016/j.cca.2019.09.009 31526774

[23] Chalasani N Younossi Z Lavine JE Diehl AM Brunt EM Cusi K. The diagnosis and management of non-alcoholic fatty liver disease: practice Guideline by the American Association for the Study of Liver Diseases, American College of Gastroenterology, and the American Gastroenterological Association. Hepatol (Baltimore Md) (2012) 55(6):2005–23. 10.1002/hep.25762 22488764

[24] Brunt EM Kleiner DE Wilson LA Belt P Neuschwander-Tetri BA. Nonalcoholic fatty liver disease (NAFLD) activity score and the histopathologic diagnosis in NAFLD: distinct clinicopathologic meanings. Hepatol (Baltimore Md) (2011) 53(3):810–20. 10.1002/hep.24127 PMC3079483 21319198

[25] European Association for the Study of the L European Association for the Study of D. European Association for the Study of O. EASL-EASD-EASO Clinical Practice Guidelines for the management of non-alcoholic fatty liver disease. J Hepatol (2016) 64(6):1388–402. 10.1016/j.jhep.2015.11.004 27062661

[26] Kleiner DE Brunt EM Van Natta M Behling C Contos MJ Cummings OW. Design and validation of a histological scoring system for nonalcoholic fatty liver disease. Hepatol (Baltimore Md) (2005) 41(6):1313–21. 10.1002/hep.20701 15915461

[27] Hosmer DW Jr Lemeshow S Sturdivant RX. Applied logistic regression. 3 ed. Hoboken, NJ: Wiley (2013).

[28] Bell LN Theodorakis JL Vuppalanchi R Saxena R Bemis KG Wang M. Serum proteomics and biomarker discovery across the spectrum of nonalcoholic fatty liver disease. Hepatol (Baltimore Md) (2010) 51(1):111–20. 10.1002/hep.23271 PMC2903216 19885878

[29] Ooi GJ Burton PR Doyle L Wentworth JM Bhathal PS Sikaris K. Modified thresholds for fibrosis risk scores in nonalcoholic fatty liver disease are necessary in the obese. Obes Surg (2017) 27(1):115–25. 10.1007/s11695-016-2246-5 27220852

[30] Tarantino G Citro V Capone D. Nonalcoholic Fatty Liver Disease: A Challenge from Mechanisms to Therapy. J Clin Med (2019) 9(1). 10.3390/jcm9010015 PMC7019297 31861591

[31] Singh S Allen AM Wang Z Prokop LJ Murad MH Loomba R. Fibrosis progression in nonalcoholic fatty liver vs nonalcoholic steatohepatitis: a systematic review and meta-analysis of paired-biopsy studies. Clin Gastroenterol Hepatol Off Clin Pract J Am Gastroenterol Assoc (2015) 13(4):643–54.e1-9; quiz e39-40. 10.1016/j.cgh.2014.04.014 PMC4208976 24768810

[32] McPherson S Hardy T Henderson E Burt AD Day CP Anstee QM. Evidence of NAFLD progression from steatosis to fibrosing-steatohepatitis using paired biopsies: implications for prognosis and clinical management. J Hepatol (2015) 62(5):1148–55. 10.1016/j.jhep.2014.11.034 25477264

[33] Zeng MD Fan JG Lu LG Li YM Chen CW Wang BY. Guidelines for the diagnosis and treatment of nonalcoholic fatty liver diseases. J Digest Dis (2008) 9(2):108–12. 10.1111/j.1751-2980.2008.00331.x 18419645

[34] Hashimoto T Cui CY Schlessinger D. Repertoire of mouse ectodysplasin-A (EDA-A) isoforms. Gene (2006) 371(1):42–51. 10.1016/j.gene.2005.11.003 16423472

